# Use of Selected Lactic Acid Bacteria and Quinoa Flour for Manufacturing Novel Yogurt-Like Beverages

**DOI:** 10.3390/foods7040051

**Published:** 2018-04-01

**Authors:** Anna Lorusso, Rossana Coda, Marco Montemurro, Carlo Giuseppe Rizzello

**Affiliations:** 1 Department of Soil, Plant, and Food Science, University of Bari “Aldo Moro”, 70126 Bari, Italy; a.lorusso@uniba.it (A.L.); montemarco@yahoo.it (M.M.); 2 Department of Food and Nutrition, Helsinki Institute of Sustainability Science, University of Helsinki, 00100 Helsinki, Finland; rossana.coda@helsinki.fi

**Keywords:** quinoa, lactic acid bacteria, beverage, fermentation, *Lactobacillus plantarum*, *Lactobacillus rhamnosus*, yogurt-like

## Abstract

This study aimed at investigating the suitability of quinoa for making yogurt-like beverages. After the selection of the adequate technological parameters, the fermentation was carried out by using different lactic acid bacteria strains: a probiotic (*Lactobacillus rhamnosus* SP1), an exopolysaccharides (EPS)-producing (*Weissella confusa* DSM 20194), and one isolated from quinoa (*Lactobacillus plantarum* T6B10). During the 20 h of fermentation, *W. confusa* caused the highest viscosity increase. All the strains had improved concentration of free amino acids and γ-Aminobutyric acid (GABA), polyphenols availability, antioxidant activity (up to 54%), and protein digestibility. The nutritional index (NI) was the highest when *L. rhamnosus* SP1 was used. The starch hydrolysis index in vitro ranged from 52 to 60. During storage at 4 °C, viscosity and water holding capacity decreased with the exception of the beverage fermented with *W. confusa*, while all the nutritional characteristics remained stable or slightly increased. Sensory analyses showed that beverages had good textural and organoleptic profiles. Besides the well-known positive properties of the raw matrix, fermentation allowed the obtainment of beverages with different features. Due to the nutritional and functional characteristics conferred to the quinoa beverages, the use of the probiotic and EPS-producing strains showed adequate potential for the industrial application.

## 1. Introduction

Amid the large number of novel and innovative functional foods under investigation or already present in the market, beverages are considered the most promising category because of convenience and possibility to meet consumer’s demands for container size, shape, and appearance, and offer great opportunities to incorporate desirable nutrients and bioactive compounds [1]. Several types of commercial functional beverages are today available and there is an increasing interest, in combination with the growing trend of vegetarianism, towards non-dairy beverages made with vegetables, fruits, and cereals [1,2,3]. The market increase is also related to the lactose intolerance/malabsorption and to the cholesterol contained in the dairy products [4]. Non-dairy traditional beverages mainly based on cereals have long existed all over the world (such as boza, bushera, chhang, chica, haria, mahewu, omegisool, pozol, togwa) [3]. In addition to these, several new non-dairy probiotic beverages have been recently developed [5]. Beyond the most common cereals, minor cereals like oat or spelt [6,7], legumes, and pseudocereals (e.g., quinoa, buckwheat, and amaranth) have been investigated as raw ingredients for making functional beverages [8,9,10]. In the last decade, the use of pseudocereals increased not only in special diets of people allergic to cereals, but also in healthy diets [11]. Quinoa (*Chenopodium quinoa*) is a pseudocereal native to the Andean regions of South America [12,13,14]. It is rich in proteins with a high biological value, carbohydrates of low glycemic index, phytosteroids, ω-3 and -6 fatty acids, and dietary fiber [11] (Kürşat Demir, 2014). Due to its nutritional quality, quinoa can play a role in functional food applications. Some studies have highlighted the effect of quinoa fermentation with selected lactic acid bacteria (LAB) to enhance the features of bread [15], pasta [16], and beverages [17]. The functional benefits of bioprocessing for making traditional and novel vegetable beverages includes probiotic activity, the ability to release or synthesize bioactive compounds, and the capability to degrade anti-nutritional factors [18,19,20,21]. Overall, fermentation is a way to naturally enhance the food matrix, without the need for additives or preservatives [22]. Fermentation by LAB can improve protein digestibility [23,24] and bioaccesibility of nutrients [6]; decrease the glycaemic index [25]; extend of shelf life through the acidification [26,27]; and increase organoleptic quality of the derived beverages [19,24].

The aim of this study was to investigate the microbial, chemical, rheological. and nutritional properties of quinoa yogurt-like beverages. LAB strains displaying different properties, i.e., probiotic (*Lactobacillus rhamnosus* SP1), exopolysaccharides (EPS)-producing (*Weissella confusa* DSM 20194), or previously isolated from quinoa flour (*Lactobacillus plantarum* T6B10) [15], were used as starters for fermentation. Analyses were carried out before and after microbial fermentation and during storage at 4 °C.

## 2. Materials and Methods

### 2.1. Raw Materials and Microorganisms

Organic quinoa flour was purchased from Food for All (Pescantina VR, Italy). The characteristics of quinoa flour indicated by the manufacturer were as follows: moisture, 10%; protein (N × 5.70), 15% of dry matter (d.m.); fat, 7% of d.m.; total carbohydrates, 75% of d.m. (sugars 6.3% and dietary fibers 9% of d.m.); ash, 2% of d.m.

*Lactobacillus plantarum* T6B10, previously isolated from a quinoa type I-sourdough [15]; *Lactobacillus rhamnosus* SP1, a commercial probiotic strain (supplied by Sacco Srl, Cadorago, CO, Italy), and the EPS-producing strain *Weissella confusa* DSM 20194 (supplied by DSMZ Collection of Microorganisms), were used as starters for fermentation.

Strains were routinely propagated at 30 °C in MRS broth (Oxoid, Basingstoke, Hampshire, United Kingdom) according to the isolation media [15] and cultivation conditions described in the DSMZ catalog.

When used for quinoa fermentation, LAB were cultivated until the late exponential phase of growth was reached (ca. 10 h), harvested by centrifugation at 9000× *g* for 10 min at 4 °C, washed twice in 50 mM phosphate buffer (4 °C, pH 7.0), and resuspended in the tap water used for making beverages.

### 2.2. Yogurt-Like Beverages

Aiming at the selection of the optimal ratio quinoa flour:water, the viscosity of mixtures including flour ranging from 20 to 50% *wt*/*wt* was determined and compared to that of commercial yogurt-like beverages made with oat (OATLY!, Malmö, Sweden) and soy (ALPRO SOYA, Wevelgem, Belgium). In particular, quinoa flour was mixed with water and homogenized with an Oster 6805 (Jarden Consumer Solutions Ltd., Cheadle, UK) mixer. Then, aliquots of 250 g of each mixture were treated at 63 °C for ca. 19 min until the inside temperature was between 54–62 °C, aiming at starch gelatinization, as described by Lindeboom, Chang, Falk & Tyler [28]. Viscosity was determined on samples adapted at 23 °C for 30 min. The apparent viscosity [29] was measured on 50 g of the mixtures using a rheometer (Anton Paar GmbH, RheolabQC, Germany) equipped with a cylinder measuring system CC27.

Based on the above results, the content of 35%, *wt*/*wt* of quinoa flour in water was selected. Three quinoa fermented beverages were obtained: B-SP1 and B-T6B10, inoculated with *L. rhamnosus* SP1 and *L. plantarum* T6B10 respectively, and B-20194, containing 25% *wt*/*wt* of quinoa flour and 10% *wt*/*wt* of sucrose, inoculated with *W. confusa* DSM 20194. Sucrose was used for promoting EPS synthesis [30]. For this latter, the percentage of quinoa flour was decreased from 35 to 25% aiming at obtaining samples with the same dry matter. Moreover, preliminary tests on this beverage showed that EPS synthesis during incubation led to viscosity values comparable to those of commercial yogurt-like beverages used as references (data not shown). The inoculum of the LAB strains (initial cell density of ca. 6 log cfu/mL) was carried out after gelatinization and cooling at 30 °C. The beverages were incubated at 30 °C for 20 h. The fermentation time, in hours, was defined as the time required to reach a pH value in the range 4–5 [31,32]. After fermentation, the beverages were stored at 4 °C for 20 days. Microbial, chemical and rheological analyses were carried out before and after incubation (Initial time (Ti) and Final time (Tf), respectively), and during storage after 1, 7, and 20 days (T1, T7, and T20, respectively).

### 2.3. Microbiological Analysis

The number of presumptive LAB was estimated by plating on MRS agar media supplemented with cycloheximide (0.1 g L), (Oxoid). Plates were incubated at 30 °C for 48 h, under anaerobiosis (AnaeroGen and AnaeroJar, Oxoid). Yeasts were counted on Yeast extract-Peptone-Dextrose agar (YPD, Oxoid), supplemented with 150 ppm chloramphenicol, at 30 °C for 72 h [33]. 

### 2.4. Determination of pH, Total Titratable Acidity (TTA) and Kinetics of Acidification

The pH was determined on-line by a HI 99161 pH-meter (Hanna Instruments, Cluji-Napoca, Romania) with a food penetration probe. Total titratable acidity (TTA) was determined with a Easy Plus Titration DTI115-SC system (Mettler Toledo, Columbus, OH, USA) on 10 g of beverage dispersed in 90 mL of distilled water and expressed as the amount (mL) of 0.1 M NaOH to get pH of 8.5. Kinetics of acidification were modeled according to the Gompertz equation as modified by Zwietering, Jongeberger, Roumbouts & van’t Riet [34]:

*y* = *k* + A exp {−exp[(V_max_·e/A)(*λ* − *t*) + 1]}

where *y* is the acidification extent expressed as dpH/dt (units of pH/h); at the time *t*; *k* is the initial level of the dependent variable to be modelled (pH units); A is the difference in pH (units) between inoculation and the stationary phase (DpH); V_max_ is the maximum acidification rate expressed as dpH/h; *λ* is the length of the lag phase expressed in hours; and *t* is the time. The experimental data were modelled through the nonlinear regression procedure of the statistic package Statistica per Windows (Statsoft, Tulsa, OK, USA).

### 2.5. Organic Acids and Free Amino Acids

Water/salt-soluble extracts from beverages were prepared following the method of Weiss, Vogelmeier & Gorg [35]. An aliquot of beverage (containing 1 g of flour) was diluted with 4 mL of Tris-HCL (pH 8.8), held at 4 °C for 1 h, vortexing at 15-min intervals, and centrifuged at 20,000*× g* for 20 min. The supernatant, containing the water/salt-soluble fraction, was filtered through a Millex-HA 0.22-µm pore size filter (Millipore Co., Bedford, MA, USA) and used for analysis. Organic acids contained in the water/salt-soluble extracts were determined by High Performance Liquid Chromatography (HPLC) using an ÄKTA Purifier system (GE Healthcare, Buckinghmshire, UK) equipped with an Aminex HPX-87H column (ion exclusion, Biorad, Richmond, CA, USA), and a UV detector operating at 210 nm [36]. Total and individual free amino acids were analyzed by a Biochrom 30 series Amino Acid Analyzer (Biochrom Ltd., Cambridge Science Park, UK) with a Na-cation-exchange column (20 by 0.46 cm internal diameter) as described by Rizzello, Nionelli, Coda, De Angelis & Gobbetti [37].

### 2.6. Total Phenols and Antioxidant Activity

The 2,2-diphenyl-1-picrylhydrazyl (DPPH) radical scavenging activity was determined on the methanolic extract (ME) of quinoa flour and doughs. Five grams of each sample were mixed with 50 mL of 80% methanol to get ME. The mixture was purged with nitrogen stream for 30 min, under stirring condition, and centrifuged at 4600× *g* for 20 min. ME were transferred into test tubes, purged with nitrogen stream and stored at ca. 4 °C before analysis. The concentration of total phenols was determined as described by Slinkard & Singleton [38], and expressed as gallic acid equivalent. The free radical scavenging capacity was determined using the stable radical DPPH [37]. The scavenging activity was expressed as follows: DPPH scavenging activity (%) = [(blank absorbance − sample absorbance)/blank absorbance] × 100. The value of absorbance was compared with 75 ppm butylated hydroxytoluene (BHT), used as the antioxidant reference.

### 2.7. Water Holding Capacity, Viscosity, Total Dry Matter and Color

The apparent viscosity was measured on fermented beverages as described in 2.2. At the beginning of the analysis, the probe started to turn with an increasing speed (1/s) and every 5 s the speed was increased linearly. Water holding capacity (WHC) was measured according to the method described by Gentès, St-Gelais & Turgeon [39]. Twenty-five grams of beverages were centrifuged (210× *g* for 20 min at 4 °C) and the supernatant (expelled water) was removed and weighted. The percentage of WHC was defined according to the equation: WHC = [(Sample weight − Expelled water)/Sample weight] × 100. Total dry matter was determined on 100 mL of beverages dried at 105 °C for 24 h [40].

The chromaticity coordinates of the beverages (obtained by a Minolta CR-10 camera) were reported as color difference, ΔE*_ab_, calculated by the following equation:

$$\Delta E_{ab}=\;\sqrt{{\left(\Delta L\right)}^{2}+{\left(\Delta a\right)}^{2}+{\left(\Delta b\right)}^{2}}$$

Δ*L*, Δ*a* and Δ*b* are the differences for *L*, *a* and *b* values between sample and reference (a white ceramic plate having *L* = 93.4, *a* = −1.8 and *b* = 4.4).

### 2.8. Nutritional Characterization

The in vitro protein digestibility (IVPD) of beverages was determined by the method of Akeson & Stahmann [41] modified by Rizzello et al. [42]. The IVPD was expressed as the percentage of the total protein, which was solubilized after enzyme hydrolysis mimicking the digestion during gastrointestinal transit. The modified method AOAC 982.30a was used to determine the total amino acid profile of the digested protein fraction [43]. Amino acids were analyzed by a Biochrom 30 series Amino Acid Analyzer as described above. Since the above procedure of hydrolysis does not allow the determination of tryptophan, it was estimated by the method of Pinter-Szakács & Molnán-Perl [44]. Chemical Score (CS) estimates the amount of protein required to provide the minimal essential amino acids (EAA) pattern for adults, which was recently re-defined by FAO in 2007 [45]. It was calculated using the equation of Block & Mitchel [46]. The sequence of limiting essential amino acids corresponds to the list of *EAA*, having the lowest chemical score [46]. The protein score indicates the chemical score of the most limiting *EAA* present in the test protein [46]. Essential Amino Acid Index (EAAI) estimates the quality of the test protein, using its *EAA* content as the criterion [47]. *EAAI* was calculated according to the equation:

$$EAAI=\;\sqrt{\frac{\left(EAA_{1}\times 100\right)\left(EAA_{2}\times 100\right)\left(\ldots \right)\left(EAA_{n}\times 100\right)\left(\mathrm{sample}\right)}{\left(EAA_{1}\times 100\right)\left(EAA_{2}\times 100\right)\left(\ldots \right)\left(EAA_{n}\times 100\right)\left(\mathrm{reference}\right)}}$$

The Biological Value (BV) indicates the utilizable fraction of the test protein [47]. BV was calculated using the equation: BV = ((1.09 × EAAI) − 11.70). The Protein Efficiency Ratio (PER) estimates the protein nutritional quality based on the amino acid profile after hydrolysis. PER was determined using the equation, developed by Ihekoronye [48]:

PER = −0.468 + (0.454 × (Leucine)) − (0.105 × (Tyrosine)).

The Nutritional Index (NI) normalizes the qualitative and quantitative variations of the test protein compared to its nutritional status. NI was calculated using the equation of Crisan & Sands [49], which considers all the factors with an equal importance:

NI = (EAA × Protein (g/100 g)/100).

### 2.9. Starch Hydrolysis Index and Predicted Glycaemic Index

The analysis of starch hydrolysis was carried out on beverages with a procedure mimicking the in vivo digestion of starch [26]. The degree of starch digestion was expressed as a percentage of potentially available starch hydrolyzed at different times (30, 60, 90, 120 and 180 min). The non-linear model proposed by De Angelis et al. [26] was applied to describe the kinetics of starch hydrolysis. The hydrolysis curves were obtained with the software Statistica 8.0. Wheat flour bread (WB) was used as the control to estimate the hydrolysis index (HI = 100). The predicted GI [50] was calculated using the equation, with wheat bread as the reference (GI wheat bread = 100): GI = 0.549 × HI + 39.71.

### 2.10. Sensory Analysis

The protocol for sensory analysis [51,52] considered a vocabulary for odor and flavor attributes. Ten trained panelists were involved in the analysis. The evaluation of sensory attributes was discussed with the assessors during the introductory training sessions. References that could be used to remind panelists about the quality of each attribute were identified (Table 1).

A scale from 0 to 10 was used. In particular, the score was attributed based on the intensity of the perception (0 = no perception; 5 = perceptible attribute; 7 = moderate perception; 10 = strong perception). With the exception of artificial, earthy and particles, none of the other attributes can be considered as negative if the perception was moderate (mean score lower than 8.5), but peculiar of the beverage. High score for uniformity of mass and adherence to spoon are considered as positive characteristics. The descriptive sensory analysis was carried out once the training was completed. Beverages were served in white polystyrene cups (40 mL in a 120 mL cup), and were labeled randomly with selected codes. Beverages were served at room temperature (20 °C) to better differentiate odors and flavors, and to facilitate the characterization and comparison of each sample. Each assessor received 2 samples for each beverage; 3 independent experiments were carried out.

### 2.11. Statistical Analysis

Beverages were produced in triplicate and all the chemical and physical analysis were carried out in triplicate for each batch of beverages. Data were subjected to one-way ANOVA; paired-comparison of treatment means was achieved by Tukey’s procedure at *p <* 0.05, using the statistical software Statistica 8.0 (StatSoft Inc., Tulsa, OK, USA).

## 3. Results

### 3.1. Beverage Manufacturing and LAB Fermentation

The viscosity values of the commercial oat and soy yogurt-like beverages were 0.455 and 0.491 Pa·s; respectively. The use quinoa flour at percentages ranging from 20 to 50% corresponded to viscosity values of the mixtures (after gelatinization) from 0.113 to 1.20 Pa·s. In particular, when quinoa flour was mixed at 35% in water, viscosity after gelatinization was 0.391 Pa·s.

The kinetics of acidification of the strains *L. rhamnosus* SP1, *L. plantarum* T6B10, *W. confusa* DSM 20194, when inoculated in the quinoa mixture, were investigated. The highest variation of pH (ΔpH) was found for *L. plantarum* T6B10 (2.35), while *L. rhamnosus* SP1 and *W. confusa* DSM 20194 gave similar lower values (Table 2).

*L. plantarum* T6B10 had also the lowest lag phase, *λ* (0.22). No significant differences (*p* > 0.05) were found for the maximum acidification rate Vmax, ranging from 0.15 to 0.18 dpH/h. The cell number of LAB at the end of fermentation was ca. 2 logarithmic cycles higher than Ti (Table 3).

During 20 h of fermentation the pH of the beverages decreased from ca. 6 up to 3.9 (Table 2). The lowest value was found in the beverage fermented with *L. plantarum* T6B10, while *W. confusa* caused the lowest pH drop (Table 3). TTA significantly differentiated the beverages and was inversely correlated to pH values. The concentration of organic acids increased as the consequence of the LAB fermentation, nevertheless, the amount of lactic acid in B-SP1 and B-20194 during fermentation was significantly lower compared to *L. plantarum* T6B10 (Table 3). Generally, acetic acid concentration was very low in all the beverages and its highest amount was found in B-20194. (Table 3). The concentration of organic acids increased as the consequence of the LAB fermentation, nevertheless, the amount of lactic acid in B-SP1 and B-20194 during fermentation was significantly lower compared to *L. plantarum* T6B10 (Table 3).

**Figure 1:**
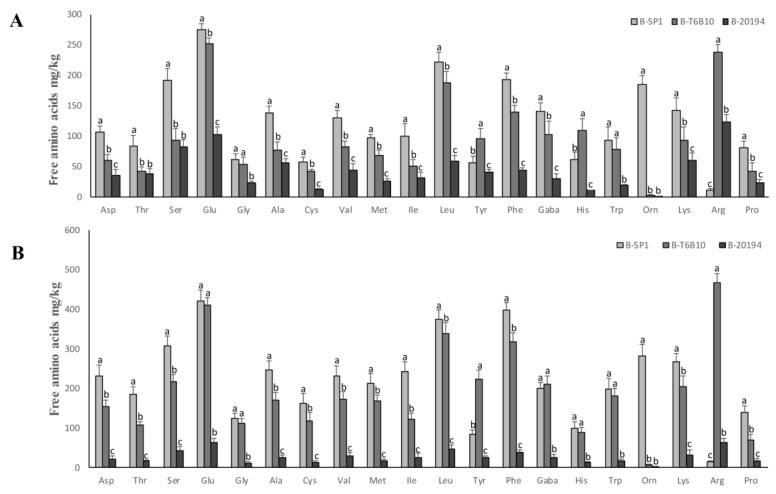
Concentration of free amino acids and their derivatives (mg/kg) of the quinoa beverages B-SP1 and B-T6B10 (containing 35%, *wt*/*wt* of quinoa flour in water), inoculated respectively with *L. rhamnosus* SP1 and *L. plantarum* T6B10, and B-20194 (containing 25% *wt*/*wt* of quinoa flour and 10% wt/wt of sucrose), inoculated with *W. confusa* DSM 20194, fermented at 30 °C for 20 h, before (**A**), and after storage at 4 °C for 20 days (**B**). Data are the means of three independent analyses. Three-letters amino acid code (IUPAC) is used. ^a–c^ Values with different superscript letters within the same amino acid, differ significantly. The error bars indicate standard deviation.

Overall, acetic acid concentration was very low in all the beverages and its highest amount was found in B-20194. (Table 3). The highest concentration of total free amino acids (FAA) was found in B-SP1 (2550 mg/kg) followed by B-T6B10 and B-20194 (Table 3). In particular, Glu, Leu, Ser, Phe and Orn were the FAA in higher quantity in B-SP1. B-T6B10 contained relevant concentration of Arg (237 mg/L) (Figure 1A). GABA, initially present at approximately 20 mg/L, increased above 100 mg/L in B-SP1 and B-T6B10 at Tf (Figure 1A).

### 3.2. Total Phenols and Antioxidant Activity

Methanolic extract (ME) from beverages were used to determine total phenols and the antioxidant activity [53]. The concentration of total phenols of B-T6B10 was significantly higher than those found in ME of B-SP1 and B-20194 (Table 3). The antioxidant activity was assayed on DPPH radical. Under the assay conditions, 100% of activity corresponds to the complete scavenging of DPPH radical (50 μM) after 10 min of incubation with the antioxidant compounds. The activity of all ME was lower than BHT (78%), used as the positive control and, similarly to the previous assay, the highest value was found for B-T6B10 while it was significantly lower in B-20194 and B-SP1.

### 3.3. Technological Characterization

The dry matter of the beverages made with the 35% of quinoa flour significantly differed from B-20194, made with 25% of flour and added of 10% sucrose (Table 3). This also influenced the initial WHC of the beverages being of 70% for B-SP1 and B-T6B10 and slightly below for B-20194 (63%) (Table 3). After 20 h of fermentation, the value of WHC of B-SP1 and B-T6B10 beverages decreased significantly; on the contrary, it markedly increased in B-20194 as the consequence of EPS production. The same trend was observed for the viscosity of beverages, since only in B-20194 the value increased during fermentation (Table 3). The three beverages showed very slight differences for the chromaticity coordinates *L*, *a*, and *b*, and similar color difference (ΔE) value (27.7–28.7) (Table 3).

### 3.4. Shelf-Life Assessment

**Figure 2:**
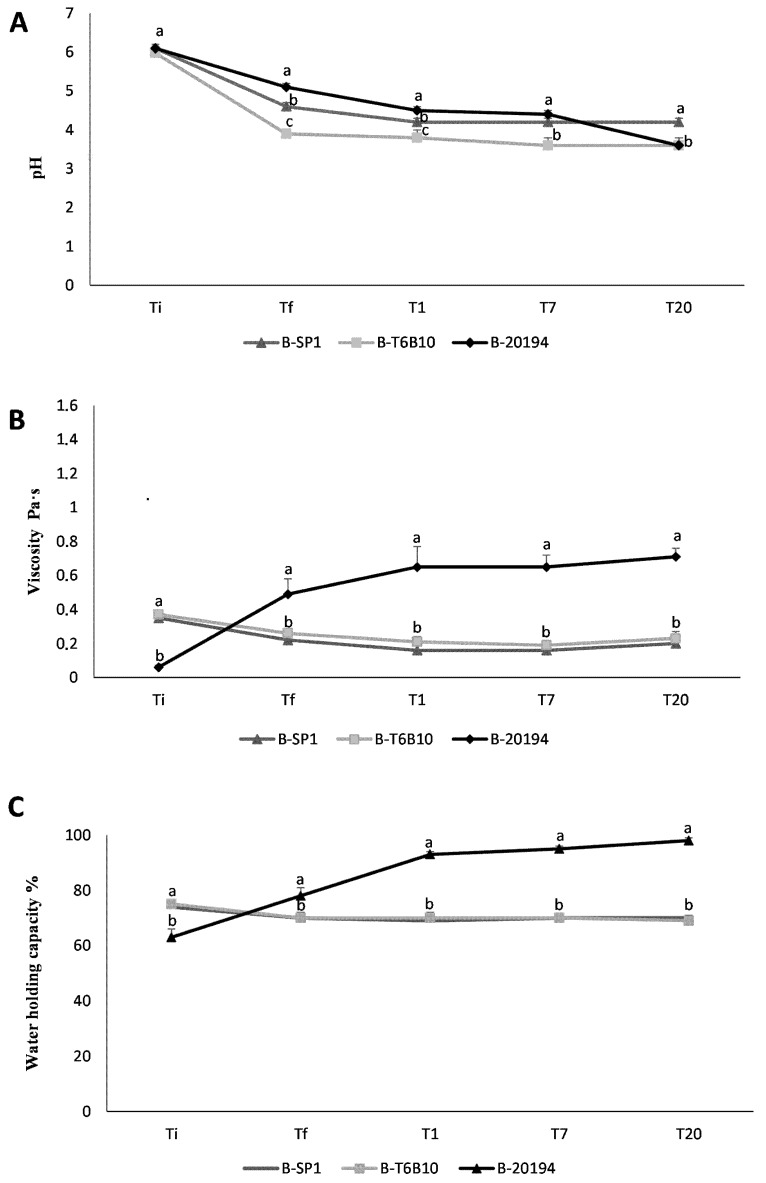
pH (**A**), viscosity (**B**), and water holding capacity (**C**) of the quinoa beverages B-SP1 and B-T6B10 (containing 35%, *wt*/*wt* of quinoa flour in water), inoculated respectively with *L. rhamnosus* SP1 and *L. plantarum* T6B10, and B-20194 (containing 25% *wt*/*wt* of quinoa flour and 10% *wt*/*wt* of sucrose), inoculated with *W. confusa* DSM 20194 determined before (Ti) and after fermentation at 30 °C for 20 h (Tf), and after 20 days of storage at 4 °C (T20). ^a–c^ Values with different superscript letters, differ significantly. The error bars indicate standard deviation.

The cell density of the LAB during the 20 days-storage remained constant and always higher than 8.5 log cfu/mL (Table 3). As expected, a further acidification was observed in all the beverages compared to Tf. The highest pH decrease during storage was observed for B-20194 after seven days (Figure 2A); nevertheless, the final value was similar to B-T6B10.

Indeed, the organic acids concentration slightly, but significantly increased during the storage (Table 3). Although lactic acid content was markedly lower in B-SP1 and B-20194 compared to B-T6B10, the final amounts observed (T20) were almost double compared to the corresponding Tf. Acetic acid concentration was the highest in B-2016, while B-SP1 was characterized by the lowest value. The concentration of TFAA markedly increased during storage in B-SP1 and B-T6B10, while only slightly in B-20194 (Table 3). Considering the individual FAA, Glu, Leu, Phe, Lys were found at the highest concentrations in B-SP1 and B-T6B10, and mainly differed for Orn (markedly higher in B-SP1) and Arg (markedly higher in B-T6B10) (Figure 1B).

According to total phenols concentration detected in ME, the antioxidant activity of the beverages further increased during storage. B-20194, produced with a lower percentage of quinoa flour, showed the lowest values (Table 3).

Viscosity slightly decreased in B-SP1 and B-T6B10, while it remained stable in B-20194 (Figure 2B and Table 3), in which the highest value was observed at T1. B-20194 also had stable WHC throughout storage time, unlike in the other beverages (Figure 2C and Table 3). The colorimetric coordinates did not show significant changes during the storage period considered (Table 3).

### 3.5. Nutritional Characterization

A multi-step protocol, which mimics the in vivo digestion, was used to estimate the beverages IVPD. Before fermentation (Ti), IVPD was ca. 71%, and it increased up to 80–86% after fermentation. B-20194 showed the lowest value, while no significant differences were observed between the other two beverages. As consequence of a moderate proteolysis occurring during the storage period, a further increase of the digestibility was found at T20 for all the beverages (Table 4).

The digestible protein fraction was further characterized and the amino acid composition and the related chemical scores were calculated. Chemical Score (CS) estimates the amount of protein required to provide the minimal essential amino acids (*EAA*) pattern for adults, which was re-defined by FAO (Food and Agriculture Organization) in 2007 [45]. Based on CS, the sequence of limiting amino acids and the protein score were determined. At T0, Lys, Cys, and Trp were the most limiting amino acids, while after fermentation, and at the end of storage, Cys, Val, and Lys, were limiting for all the beverages (Table 4). The protein score increased from 21 to 37% after fermentation, showing similar values for B-SP1 and B-T6B10, and slightly lower value for B-20194 (Table 4). The same trend was observed for *EAAI*, PER, and BV indexes, which are commonly used to estimate the quality of food proteins. All these indexes increased up to 20% from T0 to Tf, and kept increasing during the storage period.

The Nutritional Index (NI), which is affected by the amount of digested protein and the EAA ratio, ranged from 2.2 (B-20194) to 5.4 (B-T6B10) (Table 4). According to the trend of the digested protein during storage, slight increases of NI were found at T20 compared to Tf (Table 4).

Starch hydrolysis, a presumptive measure of the glycemic index (GI) in healthy subjects [25], was determined to mimic the in vivo digestion. After 180 min, the hydrolyzed starch was ca. 57% for B-SP1 and B-T6B10, and 64% for B-20194. Significant decreases of the HI (4–5%) were found for all the beverages at Tf as the consequence of the biological acidification and, for all the cases, also after the storage period (1–2%) (Table 4). Overall, no significant differences were found between the HI of B-SP1 and B-T6B10 (Table 4). The predicted GI of the beverages at Tf ranged from 68 to 75, with the highest value found for B-20194.

### 3.6. Sensory Analysis

**Figure 3:**
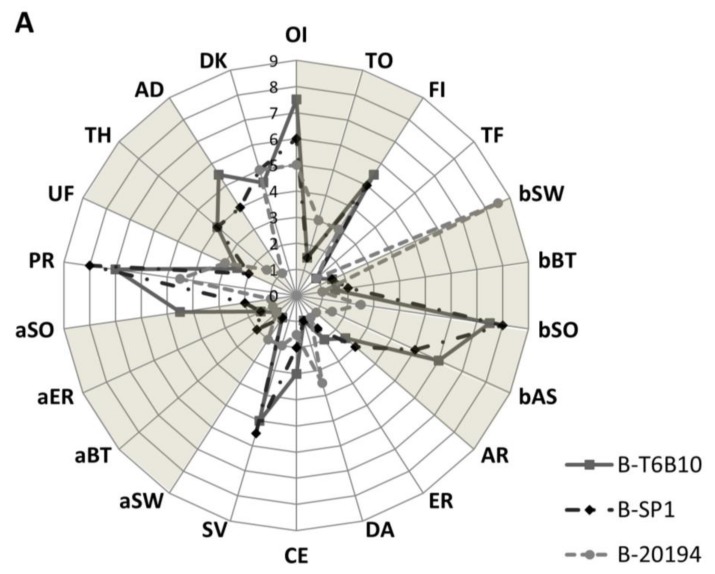

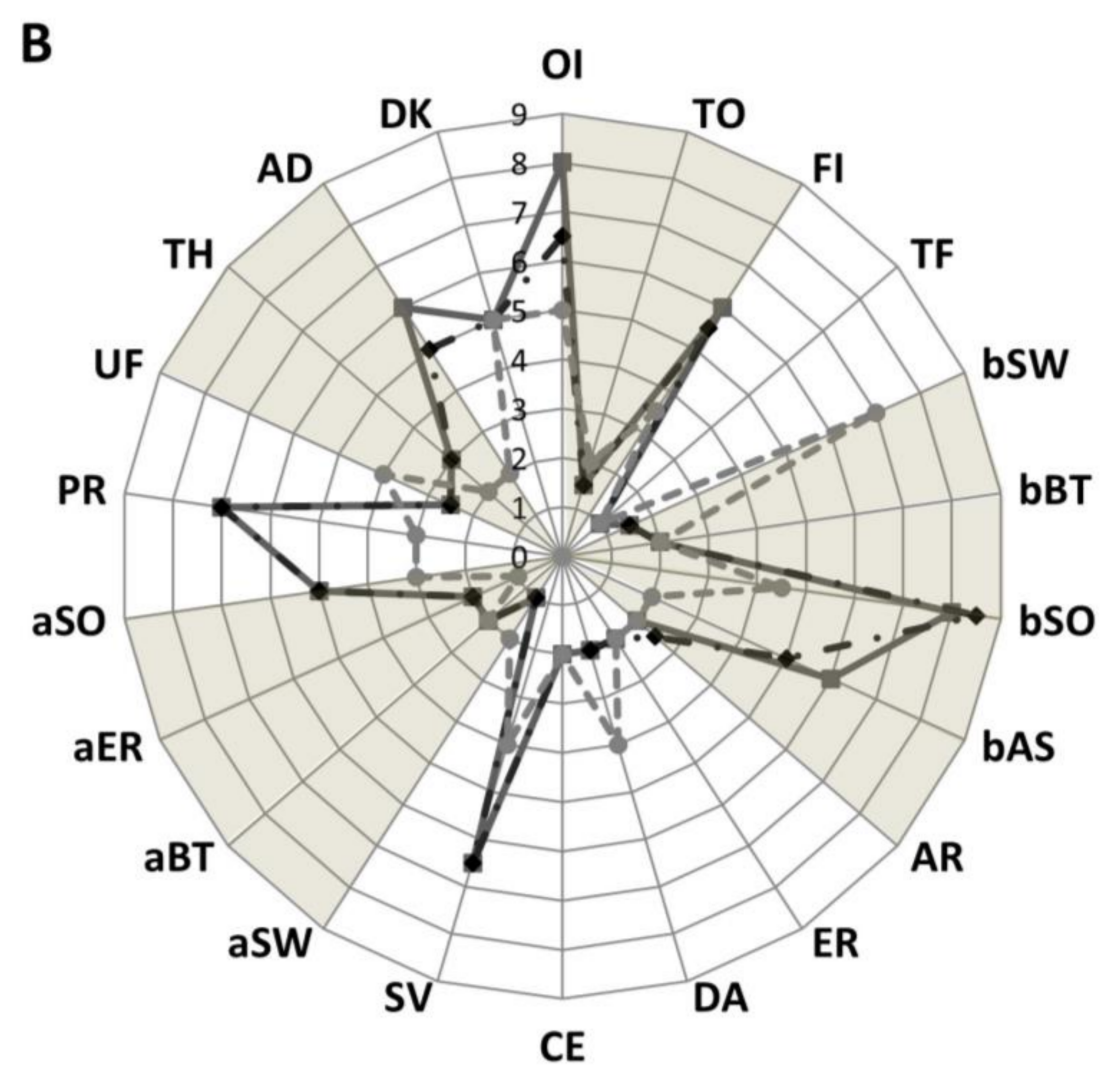
Sensory analysis of the quinoa beverages B-SP1 and B-T6B10 (containing 35%, *wt*/*wt* of quinoa flour in water), inoculated respectively with *L. rhamnosus* SP1 and *L. plantarum* T6B10, and B-20194 (containing 25% *wt*/*wt* of quinoa flour and 10% *wt*/*wt* of sucrose), inoculated with *W. confusa* DSM 20194, fermented at 30 °C for 20 h, before (**A**), and after storage at 4 °C for 20 days (**B**). The abbreviations used for sensory attributes are reported in Table 1.

Overall, the sensory profile described for B-SP1 and B-T6B10 was similar, while B-20194 received different scores for the major part of the attributes (Figure 3).

More intense odor and flavor were observed in B-T6B10. Very low scores for toasted odor and flavor were detected in all the beverages. Sweet taste characterized B-20194, together with the lowest perception of odor and astringent taste attributes. All the beverages presented low scores (<5) for artificial, earthy, dairy and cereal tastes, while savory was scored ca. 5 for B-T6B10 and B-SP1. Nevertheless, dairy score was the highest for B-20194. Scores below 5 were also received for all the aftertaste attributes (sweet, bitter and earthy), although B-T6B10 had the highest score for the sour aftertaste. The main differences in the oral texture were found for the perception of particles, higher in B-SP1 and B-T6B10 compared to B-20194, and among the manual texture descriptors, for adherence. No differences were found for appearance. After storage, sweet taste and particles perception decreased while sour and savory scores increased for all the beverages (Figure 3).

## 4. Discussion

### 4.1. Quinoa Bioprocessing through LAB Fermentation

Due to the high nutritional value, good agro-ecological adaptability and low water requirements, quinoa is a crop able to contribute to food security and farmer income as well as to the nutritional quality of the diet [54]. Quinoa is a suitable source of protein for vegetarians and vegans and, being gluten-free, it is also suitable for people suffering from coeliac disease and gluten-allergy [17]. Quinoa consumption has the potential to decrease the risk of type-2 diabetes, cardiovascular diseases, and hypertension [55,56]. Quinoa has been used for making extrudates, baked goods, and, more recently, beverages [54,57,58,59,60].

In this study, the use of quinoa as an ingredient for making a novel fermented beverage with high nutritional and functional value, was proposed. 

Fermentation with selected LAB strains isolated from quinoa allowed the increase of protein quality and digestibility and the decrease of the rate of starch hydrolysis, improving the overall quality of the fortified products such as bread and pasta [15,16]. Additionally, antioxidant compounds released by selected LAB during quinoa fermentation were previously identified and tested on human keratinocytes NCTC 2544 artificially subjected to oxidative stress [61]. The most active compounds, purified and identified as peptides having sizes from 5 to 9 amino acid residues, derived by LAB-induced proteolysis of native quinoa proteins [61]. The manufacture of traditional cereal fermented beverages or gruels takes place, in most of the cases, through spontaneous fermentation involving mixed cultures of yeasts, bacteria and fungi, and the substrates for fermentation are mainly raw or gelatinized flour, and malted grains [7,62,63].

Nevertheless, the need for proper LAB starter cultures for the fermentation of non-wheat flours has been largely recognized [19,24]. Processing with LAB would be a good option for obtaining vegetable beverages with good nutritional features and a suitable sensory profile. Moreover, the ability to synthesize oligosaccharides is a major opportunity for the development of vegetable-based prebiotic functional beverages to compete with, or replace, the existing dairy versions [6,21].

Quinoa beverages were recently developed using different processes (e.g., soaking, cooking, malting) [17,60,64,65]; however, the potential of quinoa to be used for making fermented and probiotic beverages has been only partially investigated.

In this work, three LAB strains were singly used as starters for quinoa beverages fermentation. *L. plantarum* T6B10, previously isolated from quinoa [15], showed fast adaptability to the matrix and good pro-technological characteristics (i.e., acidification kinetic and efficiency in proteolysis). During fermentation of quinoa flour, *L. plantarum* T6B10 allowed the increase of the antioxidant and phytase activities and IVPD, and the degradation of condensed tannins [15,16]. *L. rhamnosus* SP1 is a commercial probiotic strain, already employed for making emmer [7] and oat flakes beverages [6], demonstrating optimal technological properties and high survival to bioprocessing and refrigerated storage conditions. *W. confusa* has been used as a starter for different kind of fermented foods (sourdough, cereals, vegetables, fermented milk, cheeses) [66] and for its ability to produce high amounts of dextran and modify the texture in cereals [67,68,69].

The quinoa flour used in this work was obtained from seeds subjected to desaponification through washing, since pericarps containing up to 5% saponins are able to confer a bitter and astringent taste [70]. As previously reported, quinoa starch granules have very good pasting properties suitable to produce high-viscosity dough [17], characterized by excellent stability under freezing and retrogradation processes [71]. In this study, a gelatinization process was included to obtain a proper creamy texture, appreciated in similar vegetable products, and to avoid syneresis (water phase separation) during processing or storage [7]. The selection of the percentage of quinoa flour for making the beverages has taken into account the viscosity values of commercial vegetable yogurt-like products. A different ratio flour/water was used for B-20194 to promote the EPS-synthesis replacing 10% flour with sucrose [72].

### 4.2. Biochemical and Functional Characterization

The fermentation process to get a yogurt-like beverage was prolonged up to 20 h until reaching a pH value of ca. 4.0–5.0; nevertheless, the beverage fermented by *W. confusa* had less intense acidification compared to the others. During the incubation, all the LAB reached a relatively high cell density, which is a desirable functional attribute of the non-dairy beverages designed for delivering useful microorganisms [4].

*L. plantarum* T6B10, isolated from quinoa [15] showed the faster adaptation and the highest lactic acid production among the starters. The highest amount of acetic acid was found in B-20194, as consequence of the activation of the acetate kinase route due do the availability of fructose deriving from sucrose hydrolysis as external electron acceptor [73]. *W. confusa* also caused to the lower release of FAA, although the final concentration can also depend on the lower amount of quinoa flour used for making B-20194. All the free essential amino acids, including Ala and Lys, the major limiting amino acid of wheat flour [74] were 5–10 folds higher compared to a similar beverage made with oat flakes [6]. The amount of GABA found in B-T6B10 and B-SP1, up to 211 mg/kg, reached values potentially able to confer functional effects [75]. GABA is the major inhibitory neurotransmitter of the central nervous system and has several beneficial properties such as anti-hypertensive, prevention of diabetes, diuretic and tranquilizer effects [76]. 

Overall, quinoa contains a relevant amount of total phenols [15], compounds which might exert antioxidant and anti-inflammatory effects [77]. Compared to the unfermented beverages, the concentrations of total phenols in fermented beverages increased up to 61%. A similar phenomenon was already observed during quinoa fermentation [15,16]. Mostly, this is due to the combined effects of acidification, affecting solubility, and microbial hydrolytic enzymes that further promotes the release of free phenolic compounds from glycosylated and more complex forms [15,78]. As estimated towards DPPH radical [7], the increased concentration of total polyphenols observed after fermentation and during storage, corresponded to a proportional increase of the antioxidant activity.

### 4.3. Viscosity and EPS Production

As expected, and contrary to what observed for B-SP1 and B-T6B10, the viscosity and WHC of B-20194 increased during fermentation, as the consequence of the EPS synthesized by *W. confusa*, mostly of the dextran type. Recently, a strain of *W. cibaria* was employed as starter for the fermentation of a water extract from quinoa flour (“quinoa milk”), conferring good textural characteristics to the beverage thanks to the formation of a stable EPS-protein network. The exploitation of in situ synthesis of EPS is of particular interest to manufacturers of vegetable drinks aiming to imitate dairy products [24]. EPS exhibit a positive effect on the texture, mouthfeel, taste perception, and stability of fermented foods and might also have prebiotic effects [79,80,81]. Finally, EPS produced in situ can make a “natural” or “additive free” claim and comply with the requests of modern consumers [24]. 

### 4.4. Nutritional Features

The use of quinoa is important in developing countries but also encouraged in affluent countries as a substitute for refined grains such as white rice and wheat, that, if consumed in great amounts, have negative repercussions on health [17]. Proteins quality and quantity are critical components in defining the nutritional properties of food. Usually, the quality of proteins is estimated through the determination of their amino acid composition, which, in combination with protein digestibility is a predictor of the nutritive value. Fermentation with LAB caused digestibility increases in all of the beverages produced, up to 86%. The increase, already reported for different fermented matrices, is strictly related to the proteolysis phenomena during fermentation and the ability of the specific LAB used as starters. 

In this study, the digestible protein fraction was used for the determination of the protein quality indexes. It was previously defined that the total protein content analysis should hide the effect of the proteolysis, which results otherwise in similar values for samples that are instead characterized by different bioavailability and nutritional features of the protein [41]. The protein scores found for fermented quinoa beverages were markedly higher than those commonly found for wheat-based foods [15]. The *EAAI* (ratio of essential amino acids of the sample compared to the reference) and BV (the nitrogen potentially retained by human body after consumption) were the highest for B-T6B10 and B-SP1. The same was found for PER, which reflects the capacity of a protein to support the body weight gain. Within the indexes that are used to evaluate the nutritional value of foods, the NI combines qualitative and quantitative factors and is considered a global predictor of the protein quality. Since the highest protein bioavailability, the NI value of the beverages fermented with *L. rhamnosus* and *L. plantarum* (B-SP1 and B-T6B10) was more than double the value of B-20194.

HI is considered as the presumptive measure of the GI in healthy subjects [82]. It was reported that the postprandial responses to starchy foods and beverages may be modified by a number of factors, including processing conditions [83]. The disruption of the structure of the native starch by gelatinization usually increases the susceptibility to enzyme degradation, and the availability for digestion and absorption at the level of the small intestine [83]. Thermal processing causes the complete gelatinization of starch even though the rate of amylolysis could increase compared to the raw matrix [83].

Compared to wheat flour bread used as the reference, the fermented quinoa beverages had a noticeable lower value of HI (and consequently of the predicted GI values). It was recently reported that the high protein concentration (able to slow down digestion and gastric emptying) and the presence of 20-hydroxyecdysone (the most prevalent phytoecdysteroid of quinoa seeds) contribute to the lower GI of quinoa-based foods [65]. Overall, low HI is determined either by high concentration of fibers or biological acidification, which is more effective than chemical acidification [82]. It is hypothesized that the incomplete utilization of glucose and fructose deriving from the added sucrose (residual) could be responsible for the slightly higher value of the HI in B-20194 compared to the other beverages. 

### 4.5. Organoleptic Profile

Raw cereal and non-cereal flours carry very low levels of organoleptic active compounds, and in this form, give flat, “green” and unpleasant odors and flavors [19,24]. Carbohydrates, amino acids and other chemical compounds (e.g., organic acids, fatty acids) present in flours, or released from LAB as intermediate compounds during fermentation, can be channeled into different metabolic pathways that ultimately lead to specific volatile and non-volatile organoleptic compounds [19,24]. Fermentation gave to quinoa some of the typical features of a yogurt-like beverage, such as sour/acid, dairy perceptions, these last especially when the EPS-producing strain was used, without unpleasant odor, taste or aftertaste. The scores for odor/flavor intensity, together with savory confirmed an intense metabolic activity of *L. plantarum* T6B10 and *L. rhamnosus* SP1. The sweetness characterized the beverage containing EPS, together with the most appreciated textural characteristics.

### 4.6. Storage Effects

All the microbiological, chemical and technological features of the beverages were analyzed after 20 days of storage at 4 °C. LAB reached a very high cell density and, post-acidification caused a further decrease of the pH. The increased acidity was detected by sensory analysis, but all the beverages were judged acceptable also after the storage, in agreement with previous studies showing high sensory stability during shelf-life of vegetable beverages fermented with LAB [24]. Besides acidification, the moderate microbial activity occurring during storage caused the increase of FAA and antioxidant activity. Due to the progression of proteolysis, also the nutritional indexes were the highest after the 20 days of storage. Texture followed a different trend in B-SP1 and B-T6B10, characterized by a decrease in viscosity and WHC, compared to B-20194, in which it is conceivable that EPS contributed to the stability of viscosity and WHC also after 7 days of storage.

## 5. Conclusions

This study showed the suitability of quinoa flour for making functional fermented beverages. Besides the well-known positive properties of the raw matrix, fermentation with selected LAB was able to confer different qualities to the beverages. In particular, the use of the probiotic and EPS-producing strains showed adequate potential for future large-scale application.

## Figures and Tables

**Table 1:** Sensory attributes of cereal beverages, their abbreviations and descriptions.

Characteristic	Abbreviation	Definition
*Odor*		
Overall intensity of odor	OI	The odor perceived immediately
Toasted odor	TO	The odor related to toasted/coke cereal evacuate before mixing
*Flavor*		
Overall intensity of flavor	FI	The flavor evaluated orally after mixing the sample with a spoon
Toasted flavor	TF	The flavor related to toasted cereal
*Basic tastes*		
Sweet	bSW	Taste on the tongue stimulated by sugars
Bitter	bBT	Taste associated with caffeine and quinine
Sour/Acid	bSO	Taste associated with lactic acid
Astringent	bAS	Mouth drying. The complex of drying, puckering and shrinking sensations in the lower oral cavity causing contractions of the body tissue in the mouth
*Others*		
Artificial	AR	Non-specific, often used to describe imitation products
Earthy	ER	Tasting dirty and musty
Dairy	DA	A flavor of condensed, sweet milk
Cereal	CE	A flavor of cereal
Savory	SV	A meaty, fleshy, beany flavor
*After taste*		
Sweet	aSW	A lingering sweet syrupy flavor
Bitter	aBT	A lingering bitter flavor that hits the top of the tongue
Earthy	aER	A lingering dirty, earthy, musty flavor
Sour/Acid	aSO	A lingering acidic, tangy flavor
*Oral texture*		
Particles	PR	Particles presence and bits
Uniformity of mass	UF	The uniformity of mass after drink
*Manual texture*		
Thickness	TH	The force required to stir the sample with spoon
Adherence to spoon	AD	The amount of the sample adhering to the spoon evacuate by taking a spoonful of sample and turning the spoon over
Appearance		
Darkness of the color	DK	

**Table 2:** Parameters of the kinetics of acidification of the quinoa beverages B-SP1 and B-T6B10 (containing 35%, *wt*/*wt* of quinoa flour in water), inoculated respectively with *L. rhamnosus* SP1 and *L. plantarum* T6B10, and B-20194 (containing 25% *wt*/*wt* of quinoa flour and 10% *wt*/*wt* of sucrose), inoculated with *W. confusa* DSM 20194, fermented at 30 °C for 20 h.

	B-SP1	B-T6B10	B-20194
ΔpH (pH units)	1.56 ± 0.25 ^b^	2.35 ± 0.30 ^a^	1.85 ± 0.10 ^b^
Vmax (ΔpH/h)	0.17 ± 0.04 ^a^	0.18 ± 0.02 ^a^	0.15 ± 0.03 ^a^
*λ* (h)	0.93 ± 0.10 ^b^	0.22 ± 0.09 ^c^	1.71 ± 0.030 ^a^

The data are the means of three independent experiments ± standard deviations; ^a–c^ Values in the same row with different superscript letters differ significantly (*p* < 0.05).

**Table 3:** Microbiological, chemical, and technological characteristics of the quinoa beverages B-SP1 and B-T6B10 (containing 35%, *wt*/*wt* of quinoa flour in water), inoculated respectively with *L. rhamnosus* SP1 and *L. plantarum* T6B10, and B-20194 (containing 25% *wt*/*wt* of quinoa flour and 10% *wt*/*wt* of sucrose), inoculated with *W. confusa* DSM 20194 determined before (Ti) and after fermentation at 30 °C for 20 h (Tf), and after 20 days of storage at 4 °C (T20).

	B-SP1			B-T6B10			B-20194		
	Ti	Tf	T20	Ti	Tf	T20	Ti	Tf	T20
LAB cfu/mL	6.8 ± 0.1 ^f^	8.8 ± 0.1 ^d^	8.9 ± 0.2 ^d^	7.3 ± 0.1 ^e^	9.8 ± 0.1 ^a^	9.5 ± 0.1 ^b^	6.6 ± 0.1 ^f^	8.7 ± 0.2 ^d^	9.1 ± 0.1 ^c^
Yeasts cfu/mL	-	-	-	-	-	-	-	-	-
pH	6.1 ± 0.1 ^a^	4.6 ± 0.1 ^c^	4.2 ± 0.1 ^d^	5.9 ± 0.1 ^a^	3.9 ± 0.1 ^e^	3.6 ± 0.2 ^f^	6.1 ± 0.1 ^a^	5.1 ± 0.1 ^b^	3.6 ± 0.2 ^f^
TTA	5.5 ± 0.1 ^f^	12.6 ± 1 ^d^	16.5 ± 1 ^c^	5.3 ± 0.2 ^f^	18.9 ± 1 ^b^	24 ± 1.5 ^a^	4.5 ± 1 ^f^	7.8 ± 1 ^e^	14.8 ± 1 ^c^
Lactic acid (mmol/Kg)	1.3 ± 0.1 ^f^	25.7 ± 0.2 ^d^	48.8 ± 1.0 ^c^	1.4 ± 0.2 ^f^	84.37 ± 2 ^b^	115.4 ± 3 ^a^	0.3 ± 0.1 ^f^	15.36 ± 0.9 ^e^	30.6 ± 2 ^d^
Acetic acid	0.4 ± 0.2 ^e^	0.7 ± 0.2 ^d^	0.7 ± 0.3 ^d^	0.8 ± 0.1 ^d^	1.8 ± 0.5 ^c^	2.6 ± 0.8 ^b^	0.5 ± 0.1 ^d^	4.8 ± 0.9 ^a^	5.3 ± 0.7 ^a^
Total free amino acids (mg/kg)	1265 ± 40 ^e^	2550 ± 58 ^c^	4654 ± 55 ^a^	1289 ± 25 ^e^	2009 ± 64 ^d^	4067 ± 63 ^b^	776 ± 19 ^f^	1019 ± 17 ^e^	1752 ± 21 ^de^
Total phenols (mmol/kg)	5.3 ± 0.1 ^e^	5.8 ± 0.1 ^d^	9.6 ± 0.6 ^a^	5.2 ± 0.2 ^e^	8.4 ± 0.8 ^b^	9.3 ± 0.5 ^a^	4.0 ± 0.1 ^f^	5.9 ± 0.1 ^d^	7.9 ± 0.2 ^c^
Antioxidant activity	25 ± 1 ^d^	32 ± 1 ^c^	49 ± 2 ^a^	24 ± 1 ^d^	37 ± 2 ^b^	44 ± 2 ^a^	29 ± 2 ^d^	32 ± 3 ^c^	38 ± 2 ^b^
Viscosity (Pa·s)	0.35 ± 0.03 ^c^	0.22 ± 0.02 ^e^	0.20 ± 0.02 ^e^	0.37 ± 0.02 ^c^	0.26 ± 0.01 ^d^	0.23 ± 0.03 ^e^	0.06 ± 0.01 ^f^	0.49 ± 0.09 ^b^	0.70 ± 0.05 ^a^
Dry matter (g/100g)	33.3 ± 0.1 ^a^	33.3 ± 0.1 ^a^	33.4 ± 0.1 ^a^	33.9 ± 0.5 ^a^	34.2 ± 0.1 ^a^	34.4 ± 0.1 ^a^	31.1 ± 0.1 ^b^	31.6 ± 0.1 ^b^	30.3 ± 0.1 ^b^
Water holding capacity (%)	74 ± 1 ^c^	70 ± 1 ^d^	70 ± 1 ^d^	75 ± 1 ^c^	70 ± 2 ^d^	69 ± 1 ^d^	63 ± 3 ^e^	78 ± 3 ^b^	98 ± 1 ^a^
Color analysis									
*L*	65 ± 0.3 ^a^	64.7 ± 0.3 ^a^	65.1 ± 0.1 ^a^	64.8 ± 0.9 ^a^	65.0 ± 0.5 ^a^	64.9 ± 0.1 ^a^	65.5 ± 0.2 ^a^	65.6 ± 0.2 ^a^	65.7 ± 0.2 ^a^
*a*	0.2 ± 0.1 ^a^	–0.1 ± 0.1 ^a^	−0.2 ± 0.1 ^a^	0.2 ± 0.1 ^a^	−0.2 ± 0.1 ^a^	−0.23 ± 0.2 ^a^	0.2 ± 0.1 ^a^	–0.1 ± 0.1 ^a^	−0.5 ± 0.1 ^b^
*b*	8.2 ± 0.1 ^b^	8.7 ± 0.3 ^a^	9 ± 0.1 ^a^	8.3 ± 0.1 ^b^	8.3 ± 0.1 ^b^	8.1 ± 0.1 ^b^	8.2 ± 0.1 ^b^	8.4 ± 0.1 ^b^	8.2 ± 0.1 ^b^
Δ*E*	28.3 ± 0.3 ^a^	28.7 ± 0.2 ^a^	28.4 ± 0.1 ^a^	28.5 ± 0.5 ^a^	28.4 ± 0.5 ^a^	28.3 ± 0.4 ^a^	27.8 ± 0.3 ^a^	27.8 ± 0.2 ^a^	27.7 ± 0.2 ^a^

The data are the means of three independent experiments ± standard deviations; ^a–f^ Values in the same row with different superscript letters differ significantly (*p* < 0.05).

**Table 4:** Nutritional characteristics of the quinoa beverages B-SP1 and B-T6B10 (containing 35%, *wt*/*wt* of quinoa flour in water), inoculated respectively with *L. rhamnosus* SP1 and *L. plantarum* T6B10, and B-20194 (containing 25% *wt*/*wt* of quinoa flour and 10% *wt*/*wt* of sucrose), inoculated with *W. confusa* DSM 20194 determined before (Ti) and after fermentation at 30 °C for 20 h (Tf), and after 20 days of storage at 4 °C (T20).

Beverages	B-SP1			B-T6B10			B-20194		
	Ti	Tf	T20	Ti	Tf	T20	Ti	Tf	T20
In vitro protein digestibility (%)	71 ± 1 ^d^	86 ± 2 ^b^	91 ± 1 ^a^	71 ± 1 ^d^	84 ± 2 ^b^	88 ± 2 ^b^	72 ± 2 ^d^	80 ± 2 ^c^	83 ± 1 ^c^
Sequence of limiting essential amino acids (EAA)	lysine	cystine	cystine	lysine	cystine	cystine	lysine	cystine	cystine
	cystine	valine	valine	cystine	valine	valine	cystine	valine	valine
	tryptophan	lysine	lysine	tryptophan	lysine	lysine	tryptophan	lysine	lysine
Protein score (%)	22.7 ± 0.5 ^d^	30.5 ± 0.4 ^b^	34.7 ± 0.6 ^a^	22.7 ± 0.3 ^d^	31.2 ± 0.4 ^b^	33.6 ± 0.6 ^a^	22.7 ± 0.5 ^d^	27.4 ± 0.2 ^c^	29.6 ± 0.3 ^b^
Essential Amino Acid Index (EAAI)	43 ± 0.5 ^d^	47 ± 0.3 ^b^	51 ± 0.3 ^a^	43 ± 0.4 ^d^	46 ± 0.7 ^c^	50 ± 0.6 ^a^	43 ± 0.2 ^d^	46 ± 0.3 ^c^	48 ± 0.2 ^b^
Biological Value (BV)	38.3 ± 0.2 ^c^	41.3 ± 0.2 ^b^	44.3 ± 0.6 ^a^	38.3 ± 0.5 ^c^	40.5 ± 0.6 ^b^	43.8 ± 0.5 ^a^	37.8 ± 0.5 ^c^	39.5 ± 0.2 ^b^	42.8 ± 0.7 ^a^
Protein Efficiency Ratio (PER)	20.5 ± 0.6 ^c^	23.8 ± 0.5 ^b^	25.0 ± 0.2 ^a^	21.3 ± 0.4 ^c^	23.5 ± 0.2 ^b^	25.2 ± 0.4 ^a^	21.2 ± 0.1 ^c^	22.5 ± 0.2 ^c^	23.8 ± 0.6 ^b^
Nutritional Index (NI)	2.8 ± 0.1 ^c^	5.4 ± 0.2 ^b^	5.8 ± 0.2 ^a^	2.8 ± 0.2 ^c^	5.2 ± 0.3 ^b^	5.6 ± 0.1 ^a^	1.7 ± 0.3 ^e^	2.2 ± 0.3 ^d^	2.4 ± 0.4 ^c^
Hydrolysis index	57 ± 2 ^b^	52 ± 2 ^c^	50 ± 2 ^c^	57 ± 2 ^b^	53 ± 1 ^c^	52 ± 2 ^c^	64 ± 1 ^a^	60 ± 2 ^b^	58 ± 2 ^b^
Predicted GI	71 ± 3 ^b^	68 ± 1 ^c^	67 ± 2 ^c^	71 ± 4 ^b^	69 ± 2 ^c^	68 ± 2 ^c^	75 ± 4 ^a^	73 ± 4 ^b^	71 ± 2 ^b^

The data are the means of three independent experiments ± standard deviations; ^a–e^ Values in the same row with different superscript letters differ significantly (*p* < 0.05).
